# Physician-Identified Barriers to Completing the “Medical Certification for Disability Exceptions” (N-648) Citizenship Forms for Applicants with Disabilities

**DOI:** 10.1007/s10903-026-01913-z

**Published:** 2026-04-08

**Authors:** DeMarcus M. Burke, Jocelyn Ra, Mira Menon, Blessing Nnate, Shreya Salwi, Varshini Odayar, Yara S. Batista, Maya Almoussa

**Affiliations:** Medical School, University of Michigan–Ann Arbor, Ann Arbor, USA

**Keywords:** Immigrant health, Disability health, N-648, Naturalization, Immigrants with disabilities

## Abstract

Applicants for U.S. citizenship through naturalization whose disabilities prevent completion of the required English and civics examinations may submit Form N-648 (“Medical Certification for Disability Exceptions”), in which a licensed medical professional documents the limitations preventing fulfillment of these requirements. Given the central role of the N-648 in the naturalization process for applicants with disabilities and the critical role of physicians in its completion, the objective of this study was to assess physician familiarity with the N-648, perceived barriers to completion, and recommended facilitators to improve the process. We conducted a cross-sectional survey of physicians from various states and specialties that consisted of closed- and open-ended questions. Open-ended responses were reviewed and grouped into themes using an inductive approach. A total of 80 physicians were surveyed, most of whom reported limited familiarity (76%) with the N-648 yet willingness to complete the form (93.8%). Major barriers to completion included lack of clear instructions, insufficient time, and extensive documentation requirments. Physicians recommended increased training, dedicated visit times, and greater staff support as potential facilitators. Findings highlight a critical gap in physician knowledge and institutional support related to the N-648 form. Addressing these barriers through provider education, workflow adjustments, and policy guidance could improve access to disability-based naturalization accommodations and promote health equity for applicants with disabilities.

**Supplementary Information:**

The online version contains supplementary material available at https://doi.org/10.1007/s10903-026-01913-z.

## Introduction

To obtain a U.S citizenship, applicants for naturalization must demonstrate knowledge of the English language and American history in order to pass the English and civics exams [1]. The English exam assesses applicants' ability to speak, read, and write English via conversating with the interviewing officer and reading and writing one out of three sentences. The civics exam is an oral test where applicants must correctly answer 12 out of 20 questions pertinent to U.S history. Applicants have two attempts to successfully pass the exam [2]. For applicants with disabilities that impair them from learning new information or communicating their knowledge, the naturalization exam may be impossible to complete [3, 4]. The “Medical Certification for Disability Exceptions” (Form N-648) exists to ensure equitable access by exempting individuals with qualifying disabilities from these requirements [5, 6]. Per the United States Citizenship and Immigration Services, the “disability and/or impairment must result from anatomical, physiological, or psychological abnormalities” and impair the ability to “learn and/or demonstrate knowledge of English and/or U.S history and civics” [5]. For example, an applicant suffering from memory or reasoning impairments due to dementia might not be able to learn and communicate the English language or comprehend civics exam questions [7]. Additionally, the impaired functioning cannot be overcome through accomodations, such as sign-language interpreters, extended time, or off-site testing, must be expected to last more than 12 months or more, and must not be due to illegal substance use [5, 6]. The form must be completed by medical doctors, doctors of osteopathy, or clinical psychologists [6].

The success of the application hinges on several factors, such as provision of detailed medical and diagnostic documentation to prove the presence of disability and clearly explaining the “nexus,” or the connection between the applicant’s disability and inability to learn and/or demonstrate knowledge of English and/or U.S. history and civics. Completing the form with accuracy and detail is key as the credibility of the N-648 is heavily scrutinized by immigration officials, especially with the United States Citizenship and Immigration Services’ (USCIS) 2025 directive’s emphasis on safeguarding the “veracity” of the immigration pocess [4, 8, 9]. Several federal and non-profit organizations have disseminated guidance and resources on completing the N-648. While many resources describe the requirements of the N-648 and provide practical examples, important gaps remain that may affect the completion of an approvable N-648. For example, resources often fail to clarify what constitutes sufficient detail by the “preponderance of evidence” burden required by the USCIS, provide unclear examples of reasons for denial, require payment, are not specifically tailored to medical professionals, or do not mention procedural nuances (e.g., requirements for black ink or double-sided forms) [3, 4, 6, 7, 10–21]. Additionally, they often do not include detailed descriptions of the naturalization exam or available accommodations beyond those aforementioned.

Despite the critical nature of this exemption, little is known about physician familiarity with the N-648 or the challenges they face when completing it [22]. This gap in knowledge is especially concerning given the recent increase in immigration trends. In 2024, 818,500 individuals became citizens, which signifies an 12% increase in naturalization applications from pre-pandemic levels (2010–2019), which had an annual average of 730,100 newly naturalized individuals [23]. As of August 2025, it was estimated that 19,527 applicants submitted an N-648 in 2025 [24]. However, data on the types of disabilities documented in the N-648 and the proportion of immigrants with disabilities who require an N-648 are unavailable. Additionally, a recent policy change requires that the N-648 be submitted concurrently with the Application for Naturalization (N-400). There are limited exceptions, such as if a disability arises or a medical condition worsens after N-400 submission. This increases the importance of ensuring that the N-648 is completed thoroughly and accurately by healthcare providers, as applicants with new disabilities might face increased scrutiny [6, 9, 25].

To better understand physician familiarity and challenges they face in completing the N-648, we surveyed physicians across multiple specialties and states to assess their awareness of the N-648, explore considerations to completion of this form, and identify opportunities to better support providers and patients in the naturalization process. 

## Methods

### Study Design and Data Collection

We conducted a cross-sectional survey of physicians to assess familiarity with the N-648, as well as barriers and facilitators to its completion. This study was organized through the University of Michigan Asylum Collaborative, a medical student-run human rights clinic that provides free forensic medical evaluations to survivors of persecution seeking asylum in the United States. This study received exemption status by the University of Michigan IRB Committee (ID: HUM00269291).

### Participants and Recruitment

Surveys were disseminated between February 2025 and August 2025 to physicians and community organizations working with low-income and immigrant communities across several states. Recruitment strategies included distribution through chiefs of medical departments with requests to share the survey with their faculty, circulation via medical student–run asylum clinics affiliated with the Physicians for Human Rights (PHR) organization, and direct invitation to physicians attending a PHR conference. To capture diverse perspectives, no restrictions were placed on medical specialty or locations of practice. Physicians practicing in academic, community, and other healthcare settings were eligible to participate.

### Survey Measures

The survey included both closed- and open-ended questions, grouped into 4 domains:

#### Familiarity with the N-648

Physicians were asked if they were familiar with the N-648 (“Yes/No”) and to rate their familiarity from 0 (not familiar) to 5 (very familiar).

#### Patient Populations

Respondents reported whether they had worked with patients with disabilities, non-U.S. citizens (refugees, immigrants, asylum seekers), and non-U.S. citizens with disabilities. Those who had worked with non-U.S. citizens were asked if they had ever completed medical documentation for citizenship and, if so, whether this included the N-648.

#### Willingness to Complete the N-648

Respondents indicated their willingness to complete the N-648 form (“Yes”/”No”) and explained their choice through multiple-choice items with optional free-text responses. They also provided open-ended input on perceived barriers to and facilitators of N-648 completion.

#### Barriers and Recommendations

Respondents described challenges in completing the N-648 and suggested strategies that could improve feasibility, including training, institutional support, and structural changes.

### Data Analysis

Quantitative data were summarized as counts and percentages. Open-ended responses were reviewed and grouped into themes using an inductive approach. The number of responses per theme was quantified and expressed as percentages of total responses for each question. Representative quotations were selected to illustrate key themes.

## Results

### Physician Characteristics

A total of 80 physicians from 15 states responded to the survey. The majority of respondents (68.7%, *n* = 55) were from Michigan (Appendix A, Table 1). The majority (81%, *n* = 65) practiced in academic medical centers, while the remaining worked in community or other clinical settings (Appendix A, Table 2). Respondents represented a wide range of specialties, with the most represented specialty being family medicine (38.2%, *n* = 29) (Appendix A, Table 3). Responses were received between March 2025 and May 2025.

### Familiarity with the N-648

**Fig. 1:**
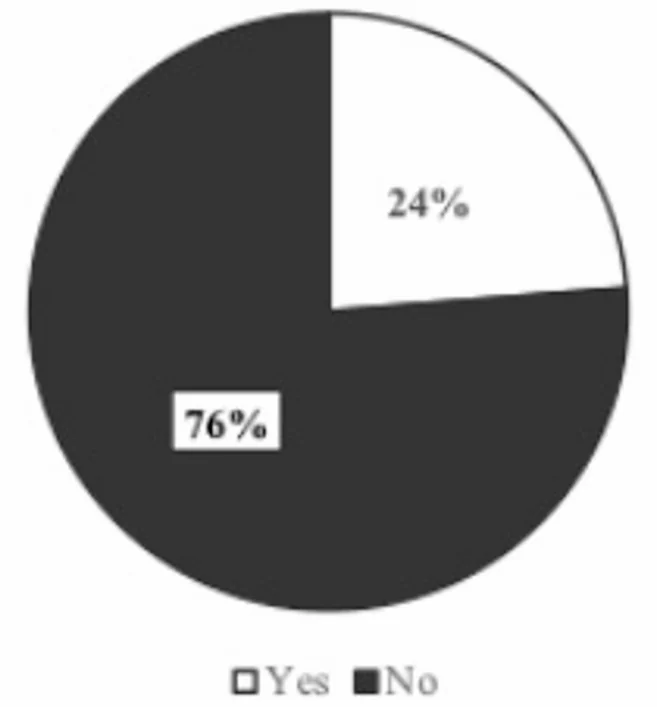
Percentage of Physicians Familiar with the N-648. Out of the 80 physicians who were surveyed, the majority (76%) reported not being familiar with the form

Most respondents (76%, *n* = 61) reported that they were not familiar with the N-648, while 24% (*n* = 19) reported familiarity (Fig. 1). Among the 19 physicians who indicated familiarity, 42% (*n* = 8) rated themselves as “quite familiar” (score of 5) (Table 1).

**Table 1:** Physician-ranked familiarity with the N-648. Physicians who indicated being familiar with the N-648 (*n*=19) were asked to rank their familiarity level from 1-5, with 1 indicating “lack of familiarity” to 5 indicating “very familiar”. The majority of respondents (42%) indicated being very familiar

Familiarity Level	Percentage of Respondents (# of Respondents)
1	0.00% (0)
2	16.0% (3)
3	16.0% (3)
4	26.0% (5)
5	42.0% (8)
Total	100% (19)

### Willingness to Complete the N-648

**Fig. 2:**
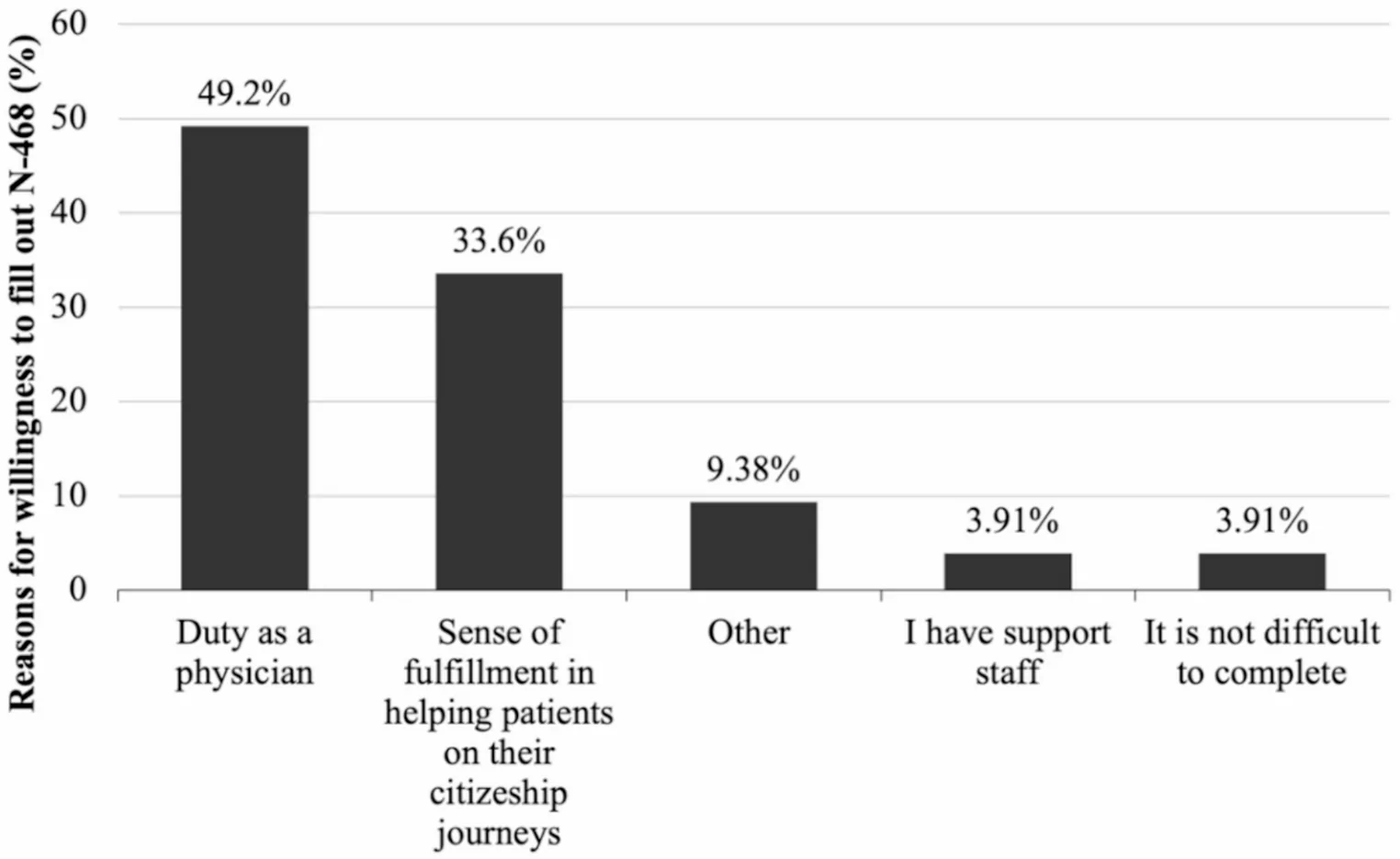
Reasons for willingness to fill out the N-648. Out of the 80 physicians surveyed, 77 physicians (96%) expressed willingness to fill out the N-648 form for patients with disabilities who are applying for U.S citizenship. The two most common reasons include the perception that it’s a physician’s duty to fill out the form and a sense of fulfillment in helping patients obtain citizenship

**Fig. 3:**
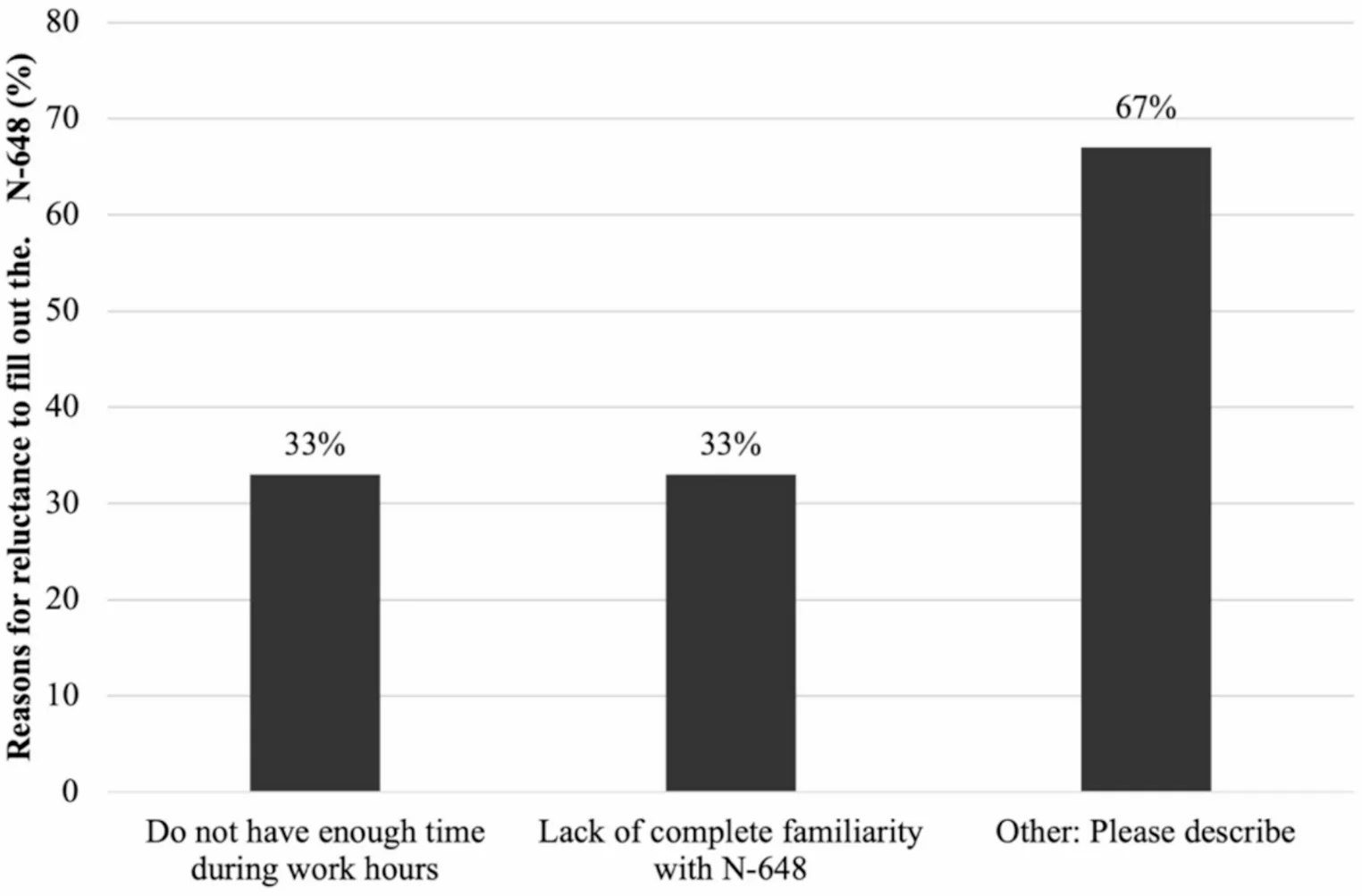
Reasons for reluctance to fill out the N-648. Out of the 80 physicians surveyed, 3 physicians (6.25%) expressed reluctance to fill out the N-648 form for patients with disabilities who are applying for U.S citizenship. Lack of familiaity with the N-648 and lack of time during work hours were cited as reasons for reluctance

Despite limited knowledge, willingness to complete the form was high: 93.8% (*n* = 75) stated they would complete the N-648 if asked. Among the 128 reasons provided for this willingness, the most common were perceiving form completion as part of their professional duty as physicians (49.2%, *n* = 63) and experiencing personal fulfillment in helping a patient’s path to citizenship (33.6%, *n* = 43) (Fig. 2). Among participants who selected “Other” (9.38%, *n* = 12), all participants provided explanations, which included having pertinent experience, considering form completion a routine part of being a physician, and having a disability themselves. In contrast, the primary reasons for reluctance included lack of familiarity with the form (33%, *n* = 1) and insufficient time to complete it (33%, *n* = 1) (Fig. 3). One of the reasons under "Other" mentioned that it is inappropriate for emergency physicians to fill out this form.

### Patient Population

All respondents (100%, *n* = 80) reported working with patients with disabilities. The majority (79%, *n* = 63) had worked with non-U.S. citizens. Two-thirds (69%, *n* = 55) had worked with non-U.S. citizens with disabilities. Of those who had worked with non-U. S citizens, 35% (*n* = 22) had been asked to complete medical documentation for citizenship, and 82% (*n* = 18) of this subgroup had previously completed an N-648 before.

### Barriers to Completion

A total of 87 reasons were indicated as barriers to completing the N-648 form. The frequency of each barrier, along with representative quotes, is summarized in Table 2.

**Table 2:** Barriers to completing the N-648 form. A total of 87 reasons were described as barriers. Reasons were grouped into themes and represented as percentage of total responses. Representatives quotes describing each barrier are provided

Barrier	Frequency %, (Number of Responses)	Representative Quotes
Lack of familiarity with the form	54.02% (*n* = 47)	“I am unaware of why the form is needed or what I am attesting to, i.e. I do not know law of citizenship [sic].”
Lack of time during visits/workday	16.09% (*n* = 14)	“It requires significant work outside of clinical hours. It is not clear what will be successful.”“Extremely time consuming, I am overloaded with patients, it often gets rejected anyway, I have no time ever in clinic.”“Form is too long, visits are too short to gather the information and fill out the form, in my experience they often reject the form and ask you to fill it out multiple forms [sic]….”
Lack of legal knowledge/fear of error	6.90% (*n* = 6)	“Any time the legal system interfaces with the medical system the result is challenging and ambiguous…” “…fear of doing it wrong such that the patient does not attain citizenship”
Length of the form	5.75% (*n* = 5)	(See quotes under “time” above—physicians emphasized length and complexity.)
Additional testing/documentation needed	5.75% (*n* = 5)	“Form requires specific tests, records from previous providers and specialists, and validated diagnostic criteria.”
Concern form will be rejected	4.60% (*n* = 4)	“… it often gets rejected anyway…”
Patient medical literacy challenges	4.60% (*n* = 4)	“…sometimes the patients do not know their medical history well and helpful records are nearly impossible to obtain.”
Requirement for specific wording	2.30 (*n* = 2)	“Form is specific and often gets rejected for wording, requires a lot of information [sic].”

### Facilitators to Completion

A total of 67 recommendations and facilitators for completing the N-648 were identified. The frequency of each theme, along with representative quotes, is summarized in Table 3.

**Table 3:** Recommendations/facilitators that would facilitate the completion of the N-648 form among physicians. A total of 67 reasons were described. Reasons were grouped into themes and represented as percentage of total responses. Representatives quotes describing each barrier are provided

Facilitator / Recommendation	Frequency %, (Number of responses)	Representative Quotes
More instructions/training	26.9% (*n* = 18)	“A guide to completing it for physicians (recommended language, implications of different choices, etc).” “a short info on it [sic], by email or webinar or in an asylum training.”
Unsure/Not familiar enough to suggest recommendations	25.4% (*n* = 17)	“I have no idea what the form is so am not [sic] able to provide an answer.”
Additional staff support	13.4% (*n* = 9)	“It would be much easier if patients brought [sic] up in the setting of a visit or if one of my support staff could contact the patient to gather some of the initial information.”
Dedicated visit time	10.4% (*n* = 7)	“Any time a patient arrives for a scheduled office visit with the single goal of completing a form together and they have that form in their hand I consider it easy to do. In most cases the situation is less optimal (patients send in forms to be completed but don’t have visits and we don’t have access in the time frame we need, patients make an appointment but don’t have the form so we have to guess about what information I’m going to need, etc.).”
Shorter form length	7.46% (*n* = 5)	“Would always love for it to be shorter! It is much easier after the 2022 revision to the form, which did make it shorter and removed asking for specific dates (i.e., date of onset of condition) which makes it easier to get form [sic] accepted.”
Use of standardized templates	5.97 (*n* = 4)	“Templates for certain diagnosis [sic] to use for these forms and validated scores for each pertinent diagnosis”
Increased patient medical literacy	2.98 (*n* = 2)	“If there were details on the disability.”
Legal support	2.98 (*n* = 2)	“…legal counsel to review a draft of the completed N-648.”
Other systemic support	–	“Shorter form, longer visits, in-person interpreters, a court system that did not constantly challenge the expertise of medical professionals and intentionally make the process cumbersome.” “Advanced notice, dedicated visits to complete the forms, a rubric of acceptable medical conditions, legal counsel to review a draft of the completed N-648.”

## Discussion

The N-648 plays a crucial role in ensuring equitable access to citizenship for immigrants. However, the majority of physicians in our survey reported little familiarity with the form, even though many cared for patients who might require it. Many indicated they would be willing to complete it, highlighting an important space for future intervention.

Key challenges included lack of training, insufficient time during clinic visits, and fear of completing the form incorrectly. Addressing these barriers require clear educational interventions, institutional support, and structural reforms. Potential solutions include integrating N-648 training into medical trainings and/or onboarding procedures, providing protected time for form completion, and expanding access to legal support, such as by institutions providing documents explaining physician’s legal responsiblities, legal experts’ contact information, or non-profits hosting workshops with legal experts [10].

These findings mirror broader concerns about physician burden from administrative tasks [26, 27]. Per the Federal Register, medical professionals spend an estinated 2.4 hours completing the N-648, highlighting the time-consuming nature of completing the form [24]. A 2022 survey from the American Medical Association found that 60% of physicians felt overwhelmed by paperwork, highlighting the need for streamlined processes and institutional backing [28]. Future studies should evaluate the impact of structured training programs on physician confidence in completing the forms and subsequent patient outcomes.

Studies across institutions and states would prove especially useful in identifying best practices for integrating legal and medical support. Importantly, future work should incorporate patient perspectives. Incorporating both provider and patient perspectives will be key to designing better interventions and also improving patient outcomes. Additionally, future work should review USCIS records to investigate approval rates for N-400 applications when an N-468 form was filed and types and frequencies of disablities mentioned in the N-648.

This study highlights several barriers surrounding completion of the N-648 from physician perspectives, all of which impede equitable access to citizenship for patients with disabilites. Addressing these concerns in future educational and administrative initiatives would support the rights and needs of vulnerable populations seeking citizenship in the United States. As the landscape of immigration continues to evolve, further research is needed to identify best practices and strategies that can enhance the experiences of both physicians and applicants in this critical process.

## Limitations

This study has several limitations. First, most respondents practiced in academic settings, which may not reflect the perspectives of providers in community or private practice. Second, surveys did not gather data on practicing in urban versus rural settings so it was not possible to stratify data based on those geographic characteristics. Third, the majority of respondents were from Michigan, limiting generalizability to states with different immigrant populations or healthcare contexts. For example, immigrants constitute approximately 7% of Michigan’s total population, with immigrants primarily from Mexico, India, Iraq, Canada, and China [29]. In contrast, states such as California and Florida have far larger immigrant populations (27 and 22%,respectively), with different predominant countries of origin, including Cuba, Haiti, Colombia, Philippines, and Vietnam [30, 31]. Third, familiarity with the N-648 form was self-reported and may not accurately reflect competency. Finally, because most physicians had never completed an N-648 form, their insights into barriers and facilitators around completing the form are limited.

## New Contribution to Literature

As far as we are aware, this is the first study to examine familiarity with the N-648 and barriers and facilitators to completing it from the perspective of physicians, who serve as key gatekeepers in the naturalization process for migrants with disabilities. While prior research discussed patient cases and gave suggestions for completing the N-648, this study addresses a critical gap by centering clinical and structural constraints faced by physicians [22]. By identifying physician- and institutional-level barriers, our findings provide actionable insights to inform interventions, including improved physician training and institutional support mechanisms. Enhancing physician support and improving the N-648 completion process has the potential to improve naturalization outcomes and, in turn, expand access to medical and social services, thereby improving the quality of life for migrants with disabilities.

## Conclusion

The N-648 form is crucial for ensuring equitable access to citizenship for immigrants with disabilities, yet our findings highlight critical gaps in physician familiarity, training, and institutional support. While most providers expressed willingness to complete the form, limited knowledge, time constraints, and concern about making errors hinder its effective use. Addressing these challenges will require tailored interventions that equip physicians with the training, resources, and confidence to complete the form. This is critical to prevent barriers to the naturalization process and support vulnerable communities.

## Supplementary Information

Below is the link to the electronic supplementary material.

Supplementary Material 1

Supplementary Material 2

## Data Availability

All research data was generated through surveys and were analyzed by authors of this study.
