# Supplementary material for: Physician-Identified Barriers to Completing the “Medical Certification for Disability Exceptions” (N-648) Citizenship Forms for Applicants with Disabilities
Source: J Immigr Minor Health. 2026 Apr 8;28(5):1166–73. doi: 10.1007/s10903-026-01913-z (PMC13612625; doi:10.1007/s10903-026-01913-z)
Supplement: Supplementary file 1 [file 10903_2026_1913_MOESM1_ESM.docx]

**Appendix A**

Table 1. Number and percentage of physicians from U.S. states that were represented among physicians surveyed.

| **State** | **Number of Physicians** | **Percentage of Physicians (%)** |
| --- | --- | --- |
| Michigan | 55 | 68.75% |
| Massachusetts | 8 | 10.0% |
| New Hampshire | 3 | 3.75% |
| New York | 2 | 2.50% |
| California | 2 | 2.50% |
| Ohio | 1 | 1.25% |
| Illinois | 1 | 1.25% |
| Connecticut | 1 | 1.25% |
| Oregon | 1 | 1.25% |
| Maryland | 1 | 1.25% |
| Pennsylvaina | 1 | 1.25% |
| Minnesota | 1 | 1.25% |
| District of Columbia | 1 | 1.25% |
| Wisconsin | 1 | 1.25% |
| Washington | 1 | 1.25% |

Table 2. Type of practice setting that surveyed physicians work in, represented by number and percentage of physicians in each practice setting.

| **Practice Setting** | **Number of Physicians** | **Percentage of Physicians (%)** |
| --- | --- | --- |
| Academic center | 65 | 81.25% |
| Federally-qualified health center | 6 | 7.5% |
| Self-owned/private clinic | 5 | 6.25% |
| Community institution | 3 | 3.75% |
| Other | 1 | 1.25% |
| Total | 80 | 100% |

Table 3. Number and percentage of specialties represented among physicians surveyed.

| **Specialty** | **Number of Physicians** | **Percentage of Physicians (%)** |
| --- | --- | --- |
| Family Medicine | 29 | 37.7% |
| Internal Medicine | 18 | 23.4% |
| Pediatrics | 10 | 12.9% |
| Psychiatry | 7 | 9.10% |
| Medicine-Pediatrics | 4 | 5.20% |
| Emergency Medicine | 4 | 5.20% |
| Radiology | 1 | 1.30% |
| Pathology | 1 | 1.30% |
| Neurology | 1 | 1.30% |
| Otolaryngology | 1 | 1.30% |
| Surgery | 1 | 1.30% |
| Total | 77 | 100% |
