# Supplementary material for: Physician-Identified Barriers to Completing the “Medical Certification for Disability Exceptions” (N-648) Citizenship Forms for Applicants with Disabilities
Source: J Immigr Minor Health. 2026 Apr 8;28(5):1166–73. doi: 10.1007/s10903-026-01913-z (PMC13612625; doi:10.1007/s10903-026-01913-z)
Supplement: Supplementary file 2 [file 10903_2026_1913_MOESM2_ESM.docx]

**Appendix B: Survey Disseminated to Physicians**

Demographics

1. Please enter the email affiliated with your institution __________
2. What specialty do you practice? __________
3. Which city and state do you practice in? __________
4. What type of environment do you work in?
   4.1 Self-owned/private clinic
   4.2 Community institution
   4.3 Federally qualified health center
   4.4 Academic center
   4.5 Other: __________

Knowledge & Familiarity

1. Are you familiar with the N-648? ☐ Yes ☐ No
   1.1 If Yes: On a scale of 1-5 (1- not familiar at all, 5- quite familiar), please rate your familiarity with the N-648 form.
2. Have you worked with non-U.S citizens (refugees/immigrants/asylum seekers) before? ☐ Yes ☐ No
   2.1 If Yes: Has anyone ever requested that you fill out a medical document for their citizenship application before: ☐ Yes ☐ No ☐ Not Sure
   2.2 If Yes in 2.1: Have you specifically filled out an N-648 form before? ☐ Yes ☐ No
3. Have you worked with patients with disabilities before? ☐ Yes ☐ No
4. Have you worked with non-U.S citizens with disabilities before? ☐ Yes ☐ No

Attitudes

5. If asked to complete an N-648 form for your patient, would you be willing to fill it out? ☐ Yes ☐ No ☐ Other: Please Explain
   5.1 If No: Why would you not be willing to fill out the N-648?
       5.1.1 Too much paperwork
       5.1.2 Do not have enough time during work hours
       5.1.3 Do not have enough support staff
       5.1.4 Do not want to engage with legal system
       5.1.5 Lack of complete familiarity with N-648
       5.1.6 High rate of rejection of the N-648
       5.1.7 Other: Please describe.
   5.2 If Yes: Why would you be willing to fill out the form?
       5.2.1 It is my duty as a medical provider
       5.2.2 It is not difficulty to fill out
       5.2.3 It is fulfilling to help a patient with their citizenship journey
       5.2.4 I have support staff who take care of it
       5.2.5 Other: Please describe
6. Who would be responsible for completing the N-648 or other medical-citizenship documents in your clinic, office, or practice?
   6.1 Myself (physician)
   6.2 Nurse practitioner/Nurse
   6.3 Physician’s assistant
   6.4 Other: Please specify

Practices

7. What makes completing an N-648 for your patient more difficult? __________
8. What would make it easier or make you more willing to fill out this form? __________
9. If needed, may we reach out with future questions to better understand the medical process surrounding the N-648? ☐ Yes ☐ No
